# BTV-14 Infection in Sheep Elicits Viraemia with Mild Clinical Symptoms

**DOI:** 10.3390/microorganisms8060892

**Published:** 2020-06-13

**Authors:** John Flannery, Lorraine Frost, Petra Fay, Hayley Hicks, Mark Henstock, Marcin Smreczak, Anna Orłowska, Paulina Rajko-Nenow, Karin Darpel, Carrie Batten

**Affiliations:** 1 Non-Vesicular Reference Laboratories, The Pirbright Institute, Ash Road, Pirbright, Woking, Surrey GU24 0NF, UK; lorraine.frost@pirbright.ac.uk (L.F.); petra.fay@pirbright.ac.uk (P.F.); hayley.hicks@pirbright.ac.uk (H.H.); mark.henstock@pirbright.ac.uk (M.H.); paulina.rajko-nenow@pirbright.ac.uk (P.R.-N.); karin.darpel@pirbright.ac.uk (K.D.); carrie.batten@pirbright.ac.uk (C.B.); 2 Department of Virology, National Veterinary Research Institute, 24-100 Puławy, Poland; smreczak@piwet.pulawy.pl (M.S.); anna.orlowska@piwet.pulawy.pl (A.O.)

**Keywords:** bluetongue, real-time RT-PCR, infection kinetics, arbovirus

## Abstract

In 2011, Bluetongue virus serotype 14 (BTV-14) was detected in Russia during routine surveillance, and was subsequently found in a number of European countries. The strain had high sequence similarity to a BTV-14 vaccine strain. We aimed to determine the risk of this BTV-14 strain causing disease in a UK sheep breed. Four Poll Dorset sheep were infected with a Polish isolate of BTV-14 and infection kinetics were monitored over 28 days. BTV RNA was detected in EDTA blood by 4 days post-infection (dpi) and remained detectable at 28 days post-infection (dpi). Peak viraemia occurred at 6 and 7 dpi with Ct values ranging between 24.6 and 27.3 in all infected animals. BTV antibodies were detected by 10 dpi using a commercial ELISA and neutralising antibodies were detected from 10 dpi. BTV was isolated between 6 and 12 dpi. All infected sheep developed mild clinical signs such as reddening of conjunctiva and mucosal membranes, with one sheep demonstrating more overt clinical signs. Two uninoculated control animals remained clinically healthy and did not have detectable BTV RNA or antibodies. The overall mild clinical symptoms caused by this BTV-14 in this highly susceptible sheep breed were in accordance with the asymptomatic infections observed in the affected countries.

## 1. Introduction

*Bluetongue virus* (BTV) of the genus *Orbivirus*, family Reoviridae, is transmitted between vertebrate hosts by biting midges (*Culicoides* spp.) [1] and causes the economically important disease known as “bluetongue”. BTV is a serologically and genetically diverse virus with a genome comprising 10 double-stranded RNA segments that encode several structural and nonstructural proteins. BTV segment-2 encodes the most variable BTV protein (VP2) which is the primary determinant of serotype [2,3] of which 24 classical serotypes exist (BTV-1 to BTV-24) while more recently, several atypical serotypes (BTV-25 to BTV-27 and onwards) have been discovered [4,5,6]. Bluetongue occurs primarily in sheep (particularly European breeds) but also in species of deer and cattle. Since 2007, multiple BTV serotypes (BTV-1, BTV-2, BTV-3, BTV-4, BTV-8, BTV-9 and BTV-16) have circulated within the EU with concurrent financial losses as a combination of direct disease impact and/or the implemented control measures such as animal movement restrictions [7,8,9]. Although multivalent modified live vaccines have been available for many years [10], only inactivated vaccines are approved for use within the EU [11] due to the risk of vaccine transmission and the potential for clinical disease in highly susceptible sheep breeds [12,13,14]. Despite the development of novel vaccine candidates which can be modified for a number of BTV serotypes and are transmissible [15], their commercial uptake has been minimal thus far.

In November 2011, a BTV-14 strain was detected in the Smolensk region of Russia and was subsequently detected in central and eastern Europe [16,17,18]. Sequence analysis revealed that representatives of this strain had a high similarity throughout the genome to the BTV-14 reference strain on which the South African vaccine strain (RSArrrr/14, a modified live vaccine) was developed [19]. This BTV did not cause clinical symptoms in the affected herds yet was found to have spread across a wide geographical area during 2011–2012 [17]. We aimed to determine the infection kinetics of this BTV-14 strain in Poll Dorset sheep to determine the risk to a UK sheep breed and ultimately enhance our understanding of BTV pathogenesis for specific strains.

## 2. Materials and Methods

Four Poll Dorset sheep (SH01 to SH04) were subcutaneously inoculated with 1 mL of BTV-14 (POL2012/01, at a titre of 6 log_10_ TCID_50_ mL^−1^) and were directly cohoused with two uninoculated sheep that were used as negative contact transmission controls (SH05 and SH06). Animals were randomised and observed for clinical signs by independent experienced animal technicians or a veterinarian throughout the experiment and a daily clinical score was recorded for each animal across the following 11 criteria: (1) redness of eyes, (2) redness of oral and nasal mucosal membranes, (3) facial oedema, (4) salivation, (5) nasal discharge, (6) cough, (7) respiratory symptoms, (8) ulcers (oral and/or nasal), (9) food uptake (scoring of reduction), (10) behaviour changes (apathy, lethargy) and (11) reddening/bleeding of the feet (average score across all 4 feet). All signs were scored on a scale from 0.5 (very mild) to 3 (severe) in 0.5 increments. Daily body temperatures were also recorded for all animals. This experiment was carried out in accordance with the UK Animal Scientific Procedure Act (ASPA) 1986 which transposes European Directive 2010/63/EU into UK national law. This experiment was conducted in compliance with a national Project License (number 70/6798) granted by the UK Home Office to KED.

EDTA blood and serum samples were taken from the jugular vein at regular intervals (Table S1). At post-mortem, oral and nasal swabs were taken along with a number of organs (Table S1). Serum samples were analysed using an anti-VP7 ELISA (BTV Early detection ELISA, ID Vet, Grabels, France) and a neutralisation test (SNT) as per [20] for BTV-14. BTV RNA was extracted from EDTA blood samples using the MagNA Pure nucleic acid extraction platform. Real-time RT-PCR was performed using a BTV-specific segment-10 assay as described by [21] using the superscript III/Platinum Taq one-step RT-PCR kit (ThermoFisher Scientific, Paisley, UK) performed on a Stratagene Mx3005P instrument (Agilent, Stockport, UK). Virus isolation was carried out using washed blood which was subsequently sonicated and added directly to KC *Culicoides sonorensis* cells. Flasks were incubated at 26 °C for 7 days following which BTV real-time RT-PCR was used as confirmation of virus isolation by indicating successful replication.

## 3. Results

**Figure 1:**
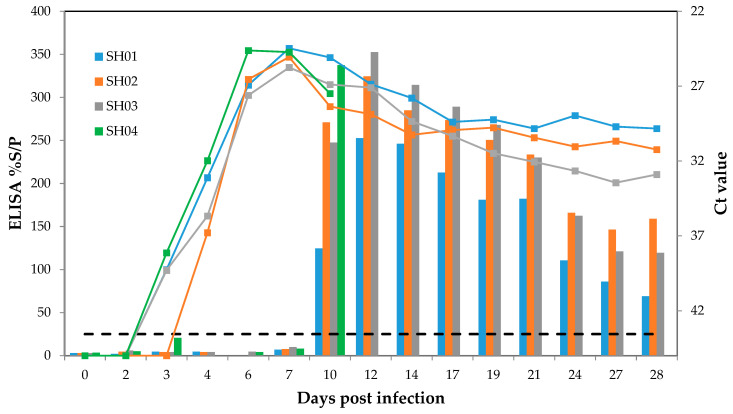
BTV RNA and antibody levels in BTV-14 infected sheep. Real-time RT-PCR Ct values are shown as coloured lines and seroconversion determined using ELISA shown as bars. The dashed line indicates the negative cut-off value of the ELISA.

Three out of four infected sheep developed mild but typical clinical signs of bluetongue such as reddening of the nasal and oral mucosa and mild facial oedema from 7 dpi until 12/13 dpi. One sheep developed slightly more overt disease in the low moderate severity including reddening of the coronary band. This sheep (SH04) was the only sheep to reach an increased body temperature of >41 °C and a daily clinical score of ≥5 for several consecutive days (Figure S1). Two infected sheep had small face ulcers on one day (7 or 8 dpi). A slightly increased rate of respiration was noted for three of the infected sheep on several occasions. BTV-14 RNA was detected in the blood of all four infected sheep by 4 dpi (Figure 1) and peak BTV-14 RNA levels in blood occurred at 7 dpi (Ct values ranging between 24.5 and 25.8). One sheep (SH04) was euthanised at 10 dpi to provide information on BTV dissemination during peak infection. In this animal at 10 dpi, BTV was detected in the lung (Ct 29.9), mandibular and retropharyngeal lymph nodes (Ct 31.1 and 28.3), spleen (Ct 26.0) and tonsil (Ct 31.1). BTV was not detected in nasal and ocular discharges and was not detected in faeces.

At the end of the experiment (28 dpi), Ct values in the remaining three infected sheep ranged between 29.8 and 32.9 in EDTA blood and BTV was detected in various internal organs (Table S1). BTV was isolated from 6 dpi to 12 dpi in all infected sheep which corresponded with peak viraemia. All four infected sheep seroconverted between 7 and 10 dpi (Figure 1). Neutralising antibodies were detected at 10 dpi (SH04), at 11 dpi (SH02 and SH03) and at 14 dpi (SH01). Neutralising antibody tires increased to 1/40 (SH01 and SH03) and to 1/60 (SH02) at the end of the experiment. The two transmission control sheep remained clinically healthy throughout the experiment with the exception of SH06 which had general reddening of mucosal membranes (eyes, mouth) at 11 dpi leading to a score of 5 for 1 day. However, both transmission control sheep did not develop a rise in body temperature, detectable BTV RNA or antibodies throughout the experiment.

## 4. Discussion

BTV outbreaks present a continuous burden to animal health which requires appropriate control responses to mitigate the associated financial losses. The improper use of modified live vaccines has been the cause of a number of European BTV outbreaks [13] which has led to the EU-wide ban on their use for BTV control [11,15]. The vaccine-derived BTV-14 represents the most recent example of improper vaccination having an impact within Europe [16]. The infection kinetics of the BTV-14 strain (POL2012/01) used in our study were similar to those of other BTV serotypes in sheep [22,23,24]. In accordance with epidemiological findings in the field, BTV-14 elicited mostly mild clinical symptoms in the sheep species we used. However, the potential of a vaccine-derived BTV strain to cause more overt clinical disease in highly susceptible breeds was reported previously [14] albeit of much milder severity in contrast with a field strain of BTV-8 in Poll Dorset sheep [25]. Furthermore, as BTV-14 was not detected in nasal and ocular discharges or faeces, nor did it transmit to the in-contact transmission control animals, we conclude that it is unlikely to be spread outside of a host–vector scenario. The recorded clinical score in one of the control sheep demonstrates that some clinical signs such as reddening of eyes are of low specificity for BTV. The BTV-14 situation was resolved by 2012 through culling and movement restrictions and had a relatively minor impact on animal health. Nevertheless, it served as a reminder of the potential for modified live vaccines to impact upon animal health and veterinary services and supports their exclusion from use to control bluetongue.

## Supplementary Materials

The following are available online at https://doi.org/10.5281/zenodo.3886529; Table S1: Mean ELISA S/P% value and real-time RT-PCR Ct values, and at https://doi.org/10.5281/zenodo.3886523; Figure S1: Clinical observations of sheep.
